# Alterations of Brain Structural Network in Parkinson’s Disease With and Without Rapid Eye Movement Sleep Behavior Disorder

**DOI:** 10.3389/fneur.2018.00334

**Published:** 2018-05-11

**Authors:** Tao Guo, Xiaojun Guan, Qiaoling Zeng, Min Xuan, Quanquan Gu, Peiyu Huang, Xiaojun Xu, Minming Zhang

**Affiliations:** Department of Radiology, The Second Affiliated Hospital of Zhejiang University School of Medicine, Hangzhou, China

**Keywords:** Parkinson’s disease, rapid eye movement sleep behavior disorder, magnetic resonance imaging, structural correlation network, graph theory analysis

## Abstract

**Background and objective:**

Rapid eye movement sleep behavior disorder (RBD) has a strong association with alpha synucleinpathies such as Parkinson’s disease (PD) and PD patients with RBD tend to have a poorer prognosis. However, we still know little about the pathogenesis of RBD in PD. Therefore, we aim to detect the alterations of structural correlation network (SCN) in PD patients with and without RBD.

**Materials and methods:**

A total of 191 PD patients, including 51 patients with possible RBD (pRBD) and 140 patients with non-possible RBD, and 76 normal controls were included in the present study. Structural brain networks were constructed by thresholding gray matter volume correlation matrices of 116 regions and analyzed using graph theoretical approaches.

**Results:**

There was no difference in global properties among the three groups. Significant enhanced regional nodal measures in limbic system, frontal-temporal regions, and occipital regions and decreased nodal measures in cerebellum were found in PD patients with pRBD (PD-pRBD) compared with PD patients without pRBD. Besides, nodes in frontal lobe, temporal lobe, and limbic system were served as hubs in both two PD groups, and PD-pRBD exhibited additionally recruited hubs in limbic regions.

**Conclusion:**

Based on the SCN analysis, we found PD-pRBD exhibited a reorganization of nodal properties as well as the remapping of the hub distribution in whole brain especially in limbic system, which may shed light to the pathophysiology of PD with RBD.

## Introduction

Rapid eye movement (REM) sleep behavior disorder (RBD) is a common parasomnia characterized by the loss of normal skeletal muscle atonia during REM sleep with prominent dreaming and motor activity (1–3). Studies showed that it has a strong association with alpha synucleinpathies such as Parkinson’s disease (PD) (4, 5), where it can either occur during the course of the disease or appear in the prodromal phase of PD (6). The prevalence of RBD in PD is up to 37–47% (7) and PD patients with RBD tend to have a poorer prognosis in terms of postural instability, gait disturbance, cognitive impairment, and even an association with visual hallucination compared with PD patients without RBD (8, 9). However, we still know little about the pathogenesis of RBD in PD, which may block our further understanding of complex substrate of PD.

Parkinson’s disease is a neurodegenerative disease characterized by alpha-synuclein accumulation, progressing in an ascending pattern from lower brainstem to limbic system and then to neocortex (10). Patients with RBD symptoms exhibited abnormalities in sleep-wake transitions (11, 12), which stem from the aberrant brainstem reticular formation (13). Alpha-synuclein accumulation in brainstem structures such as reticular formation may lead to the presence of RBD symptoms (14). Current physiological studies indicate that limbic system including amygdala and hippocampus, and neocortex such as frontal lobe play important roles in REM sleep, which is abnormal in patients with RBD symptoms (13, 15, 16). Therefore, based on the pathological substrate in brainstem, we argued that searching *in vivo* evidence would help disclose the differential phenotype of cerebral abnormalities during the clinical manifestation of RBD symptoms in PD patients.

Neuroimaging is becoming a powerful tool for investigating brain structure or function *in vivo*. To date, there have been increasing neuroimaging studies to investigate the alterations both in idiopathic RBD (iRBD) and in PD patients with RBD (PD-RBD). Increased gray matter (GM) density in hippocampus was observed in iRBD (17). PD-RBD patients showed decreased neocortical, limbic cortical, and thalamic cholinergic innervation (18), decreased regional GM volume in hippocampus and left posterior cingulate (19), decreased brain activity in primary motor cortex (20), and increased volume in frontal areas, mid-cingulate gyrus, and superior temporal gyrus (21). Taken together, we postulated that PD-RBD patients might exhibit abnormalities in whole brain including limbic system and neocortex. However, the understandings of these potential changes underlying the PD-RBD are unclear.

Recently, structural correlation network (SCN), a graph theory analysis, proposed a specific method to explore brain organization (22–24). SCN employs correlation analysis for a cross-sectional imaging data, could measure synchronized morphological alterations undergoing common pathological processes between brain regions (25, 26). Recent studies have suggested that PD patients are associated with abnormal SCN such as reduced global efficiency and reorganization of network hub in cortical thickness-based SCN (27–29). Another morphometric feature, GM volume, could also be used to construct large-scale SCN, which could reveal intrinsic structural organization in human brain (30). Considering that the alterations in PD-RBD may be widespread around the brain, therefore, in our study, we constructed GM volume-based SCN to explore the topological changes in whole brain in PD patients with possible RBD (PD-pRBD) and PD patients with non-possible RBD (PD-npRBD). We hypothesized that both PD-pRBD and PD-npRBD would exhibited disrupted structural organization and PD-pRBD would exhibited specific alterations related to limbic system or neocortex.

## Materials and Methods

### Participants

Data used in the preparation of this article were obtained from the Parkinson’s Progression Markers Initiative (PPMI) database (www.ppmi-info.org/data). For up-to-date information on the study, please visit www.ppmi-info.org. Written informed consent was obtained from all participants, and all PPMI sites received approval from their respective ethics committee on human experimentation prior to study initiation. PPMI is a large multicenter study, and consequently, a mixture of different MRI acquisitions have been used in the generation of the imaging dataset. To create a homogenous MRI dataset, we only selected those participants who had undergone T1 MRI scanning with a magnetization prepared rapid gradient echo (MPRAGE) sequence. Those PD subjects who did not have data of RBD Sleep Questionnaire (RBDSQ), MDS-UPDRS-III in off status were excluded. Normal controls (NCs) with RBDSQ score more than five were also excluded in order to remove the possibility of RBD. For a specific subject, we usually selected his baseline data, in case of poor image quality, the follow-up data were selected instead. Then, 215 PD patients and 81 NCs were initially recruited. The presence of possible RBD (pRBD, i.e., a history of dream enactment suggested by questionnaire response, but not confirmed with clinical interview/polysomnogram) was assessed by the RBDSQ (31). RBDSQ is a 10-item, patient self-rating questionnaire assessing the subject’s sleep behavior. The maximum total score of the questionnaire is 13 (32). A score of ≥6 maximizes sensitivity/specificity for RBD in the PD population and was considered as pRBD in this study (33). PD patients with a score of ≤ five of RBDSQ were classified as non-possible RBD (npRBD).

All participants’ neuropsychological performance and motor function were assessed. Participants’ neuropsychological performance was measured across a variety of cognitive tests, including Hopkins Verbal Learning Test-Revised (HVLT-R), Benton Judgment of Line Orientation (BJLO), letter number sequencing (LNS), semantic fluency (SF), and symbol digit modalities test (SDMT). Motor function for all participants in this study was evaluated using MDS-UPDRS-III and Hoehn & Yahr (H&Y) stage.

### MRI Data Acquisition and Preprocessing

All three-dimensional T1-weighted MRI data were downloaded from the PPMI database. All of these images were acquired in the sagittal plane on Siemens scanners (Erlangen, Germany) at different centers using the MPRAGE sequence. The acquisition parameters were as follows: repetition time = 2,300 ms; echo time = 2.98 ms; inversion time = 900 ms; flip angle = 9°; matrix = 240 × 256; voxel = 1 mm × 1 mm × 1 mm; and slice number = 176.

T1-weighted structural images were preprocessed using Computational Anatomy Toolbox (CAT12) (http://dbm.neuro.uni-jena.de/cat12/) with SPM 12 (http://www.fil.ion.ucl.ac.uk/spm/software/spm12/). All T1-weighted images were segmented into GM, white matter, and cerebrospinal fluid. Then, MRI inhomogeneities and noise were removed and image intensities were normalized. The resulted images were registered to the Montreal Neurological Institute standard space. A partial volume estimation was extended to account for mixed voxels with two tissue types and spatial normalization was conducted using DARTEL. Subsequently, we estimated the total intracranial volume (TIV) and then conducted quality control of all images before further analysis. To further control the image quality, we used mean correlation, Mahalanobis distance and weighted overall image quality algorithms to quantify image quality. Raw images from a noticeable lower quality (below 2 SD) were rechecked and finally excluded (24 PD patients and five NCs). Finally, the obtained preprocessed structural data from 191 PD (51 PD-pRBD and 140 PD-npRBD) patients and 76 NCs were smoothed with an 8 mm full width at half maximum.

### Construction of SCN

**Figure 1:**
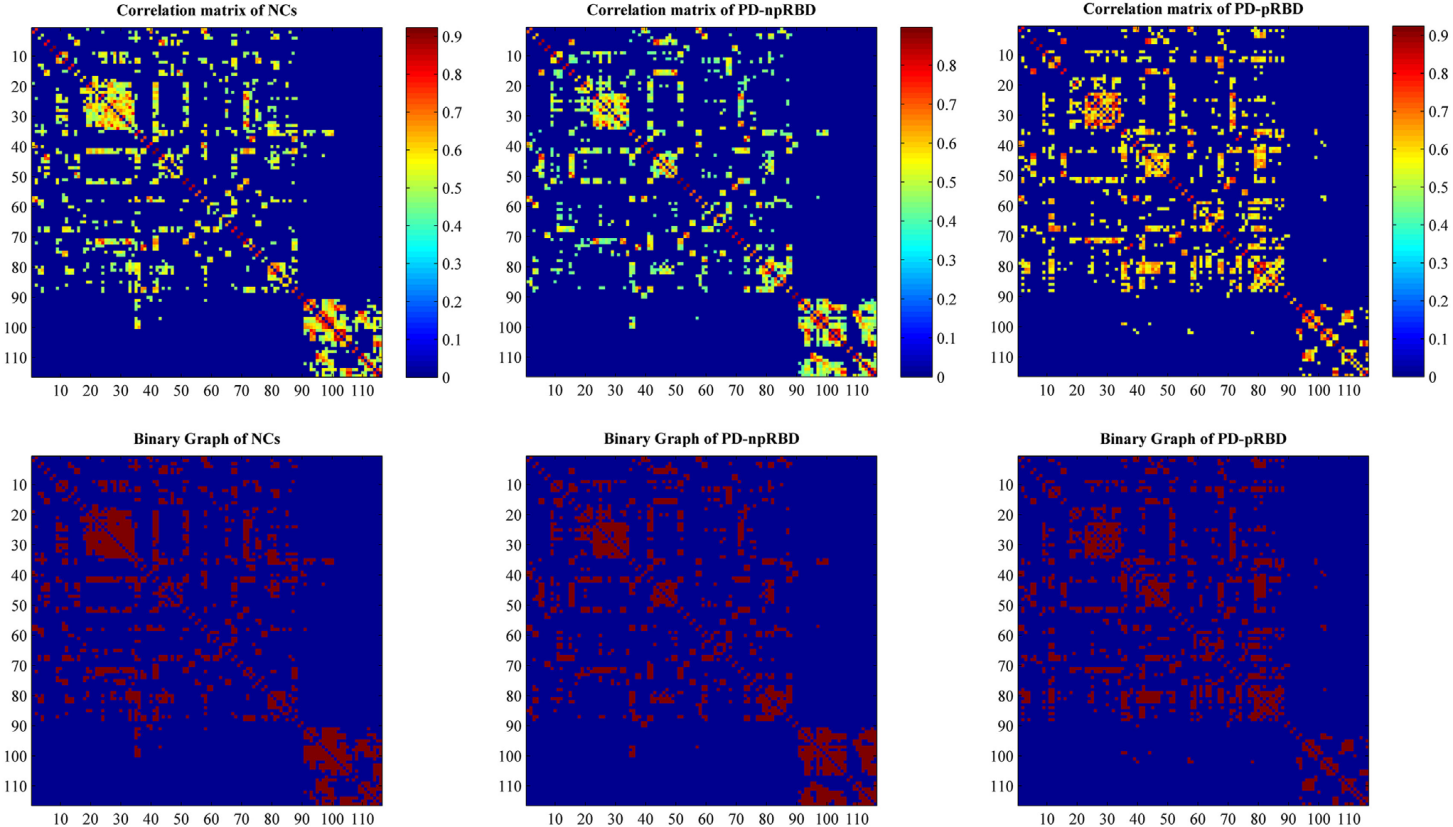
Structural correlation matrices for normal controls (NCs), Parkinson’s disease (PD) patients with possible rapid eye movement sleep behavior disorder (RBD) (PD-pRBD), and PD patients with non-possible RBD (PD-npRBD). The upper row represents weighted structural correlation matrices for NCs, PD-npRBD, and PD-pRBD; the color bar shows the strength of the connections. The lower row represents corresponding binary correlation matrices for NCs, PD-npRBD, and PD-pRBD; red color shows the presence of connection.

Individual modulated, normalized GM maps were used for graph analysis with GAT toolbox (https://mailman.stanford.edu/mailman/listinfo/gat_user_forum). Firstly, we selected 116 ROIs including bilateral cortical, subcortical and cerebellar gray created using the Automatic Anatomic Labeling atlas as nodes, and extracted volume information within each ROI for each subject. For each group, a 116 × 116 structural correlation matrix (Figure 1) was generated by performing Pearson correlation. The nuisance covariates including age, gender, and TIV were removed by performing a linear regression analysis. The edge between each pair of nodes was constructed when the correlation strength between the corresponding brain regions exceeded a certain threshold. Since thresholding, the correlation matrices at different thresholds could produce networks with different number of edges and may influence the network properties (23), we thresholded the correlation matrices at a range of network densities (from the estimated minimum density of 0.1 to maximum density of 0.5 with an interval of 0.02) ensuring the same number of edges in different networks. Then, we compared the network topologies among different groups at each level. The edges in the SCN represented the GM similarity between each pair of nodes.

### Network Analyses

#### Small-World Parameters

The small-worldness of a complex network has the following two crucial metrics: the clustering coefficient (*C*) and the characteristic path length (*L*). To evaluate the topology of the brain network, these parameters were compared to the corresponding mean values of a random graph (20 generated null networks) with the same number of nodes and edges as the network of interest. We obtained the normalized clustering coefficient (γ = *C*/*C*_rand_) and the normalized characteristic path length (λ = *L*/*L*_rand_), and thus small-worldness index (σ = γ/λ) could be computed. In a small-world network, γ > 1 and λ ≈ 1 or σ > 1, which mean that the clustering coefficient is significantly higher than that of random networks, while the characteristic path length is comparable to random networks.

#### Network Measures

We calculated the global measures including global efficiency, mean local efficiency, regional measures including nodal degree, nodal betweenness, nodal clustering coefficient, and nodal local efficiency, and hub distribution. Briefly, global efficiency measures the ability of parallel information transfer in the network, and mean local efficiency measures the fault tolerance of the network, indicating the capacity for information exchange within each subgraph when the index node is eliminated (34). Nodal degree measures the number of connections to a node, which indicates a node’s accessibility; nodal betweenness reflects the important roles of nodal communication across a node as the bridge; nodal clustering coefficient measures how close the node’s neighbors are to forming a clique; nodal local efficiency indicates the capacity of the subgraph to exchange information if a given node is eliminated (35–38). A node is considered as a hub if its nodal degree is one SD higher than the mean network degree. Detailed calculations of these network measures are shown in Supplementary Material.

### Statistical Analyses

Differences between groups in demographic and neuropsychological variables were analyzed using independent sample *T* test for normally distributed continuous data, Mann–Whitney *U* test for non-normally distributed continuous data, Pearson’s chi-squared test for categorical data in SPSS 19.0. The normality of the data was confirmed by one-sample Kolmogorov–Smirnov test. The significance of group differences was set at *P* < 0.05.

In order to test the statistical significance of the between-group differences (PD-pRBD vs. NCs, PD-npRBD vs. NCs, PD-pRBD vs. PD-npRBD) in network global topology and regional nodal measures, a non-parametric permutation test with 1,000 repetitions was used. Each network metric extracted across the specified density range (0.1:0.02:0.5) is represented by a curve that depicts the change in a specific network metric (for each group) as a function of network density. In order to compare these curves between groups, a summary measure using areas under the curve (AUC) analysis was performed. Finally, GAT generated the plots of between-group differences in regional network measures along with the quantified confidence intervals as a function of network density. *P* < 0.05 was regarded as significant.

### Validation Analyses

As the different cognitive performance between patient groups may influence brain organization, we also constructed another network in the same procedures, but with extra covariates included scores of BJLO, HVLT-R_total recall, HVLT-R_delayed recall, HVLT-R_retention, HVLT-R_recognition discrimination index, LNS, SF, and SDMT (results shown in Supplementary Material). Of note, due to the missing data of SDMT score in four PD patients (one for PD-pRBD and three for PD-npRBD), only 50 PD-pRBD and 137 PD-npRBD were included.

## Results

### Cohort Characteristics

No significant difference in age, gender, and education was found between any two groups (Table 1). PD-pRBD and PD-npRBD were not significantly different in MDS-UPDRS-III and H&Y stage. As for cognitive performance, PD-pRBD patients performed worse on all of cognitive tests compared with NCs. When comparing with PD-npRBD, PD-pRBD performed worse on most of cognitive tests except the HVLT-R recognition discrimination index. And we detected PD-npRBD performed worse in HVLT-R recognition discrimination index and SDMT compared with NCs (Table 1). After exclusion of subjects with poor image quality, data analyses were conducted in a relatively smaller sample than a previous study using PPMI database (21). Of note, the similar statistical distribution of clinical features, such as cognitive performance, indicated the sample size was still robust to detect the imaging phenotype between PD-pRBD and PD-npRBD.

**Table 1:** Characteristics of normal controls, Parkinson’s disease (PD) patients with possible rapid eye movement sleep behavior disorder (RBD) (PD-npRBD) patients and PD patients with non-possible RBD (PD-pRBD) patients.

	Controls (*n* = 76)	All patients (*n* = 191)	PD-npRBD (*n* = 140)	PD-pRBD (*n* = 51)	Controls vs. PD-pRBD (*p* value)	Controls vs. PD-npRBD (*p* value)	PD-pRBD vs. PD-npRBD (*p* value)
Age, years	60.3 (10.6)	60.9 (9.6)	60.5 (9.7)	61.9 (9.5)	0.387^a^	0.908^a^	0.364^a^
Gender, M/F	50/26	116/75	82/58	34/17	0.918^b^	0.299^b^	0.311^b^
Education, years	16.1 (2.8)	15.4 (3.0)	15.4 (3.0)	15.2 (2.7)	0.081^c^	0.114^c^	0.690^c^
MDS-UPDRS-III	0.6 (1.4)	21.3 (9.4)	20.9 (9.4)	22.4 (9.4)	**0.000^c^**	**0.000^c^**	0.338^a^
Hoehn & Yahr (H&Y) stage	–	1.6 (0.5)	1.6 (0.5)	1.7 (0.6)	–	–	0.293^b^
Total intracranial volume, cm^3^	1,553.0 (133.2)	1,590.0 (148.8)	1,593.5 (145.3)	1,580.3 (159.1)	0.298^a^	**0.045^a^**	0.589^a^
BJLO	12.4 (2.9)	12.0 (3.0)	12.4 (2.7)	11.0 (3.5)	**0.022^a^**	0.966^c^	**0.016^c^**
HVLT-R_total recall	49.0 (11.2)	47.0 (11.4)	48.1 (11.1)	43.9 (11.8)	**0.014^a^**	0.556^a^	**0.023^a^**
HVLT-R_delayed recall	49.9 (10.8)	48.9 (12.4)	51.1 (12.0)	43.2 (11.4)	**0.001^a^**	0.458^a^	**0.000^a^**
HVLT-R_retention	51.8 (9.9)	51.3 (11.7)	53.0 (11.4)	46.7 (11.4)	**0.024^c^**	0.528^c^	**0.004^c^**
HVLT-R_rec	55.5 (11.5)	50.8 (12.5)	51.4 (12.1)	49.0 (13.4)	**0.000^c^**	**0.004^c^**	0.089^c^
LNS	12.1 (2.7)	11.5 (2.8)	11.8 (2.7)	10.7 (3.0)	**0.006^a^**	0.487^c^	**0.039^c^**
SF	53.1 (9.0)	52.2 (10.2)	53.2 (10.3)	49.4 (9.3)	**0.028^a^**	0.930^a^	**0.022^a^**
SDMT	50.2 (9.7)	45.2 (9.5)	46.5 (9.0)	41.4 (10.1)	**0.000^a^**	**0.006^a^**	**0.001^a^**
GDS	5.4 (1.6)	5.3 (1.4)	5.3 (1.4)	5.2 (1.4)	0.712^c^	0.798^c^	0.580^c^
STAI	94.6 (6.9)	93.2 (8.8)	93.3 (8.3)	93.0 (10.0)	0.265^a^	0.212^a^	0.737^a^
RBDSQ	2.1 (1.5)	4.4 (2.7)	3.0 (1.3)	8.3 (1.7)	**0.000^c^**	**0.000^c^**	**0.000^c^**

*Presented by mean (SD). Bold for *p* < 0.05*.

*^a^Calculated using independent sample T test*.

*^b^Pearson’s chi-squared test for gender and H&Y stage*.

*^c^Mann–Whitney U test for education and MDS-UPDRS-III of controls and PD-pRBD/PD-npRBD*.

*Abbreviations: BJLO, Benton Judgment of Line Orientation; HVLT-R_rec, Hopkin’s Verbal Learning Test-Revised recognition discrimination index; LNS, letter number sequencing; SF, semantic fluency; SDMT, symbol digit modalities test; GDS, Geriatric Depression Scale; STAI: State-Trait Anxiety Inventory; RBDSQ: RBD Sleep Questionnaire*.

### Network Analyses

#### Small-World Parameters

**Figure 2:**
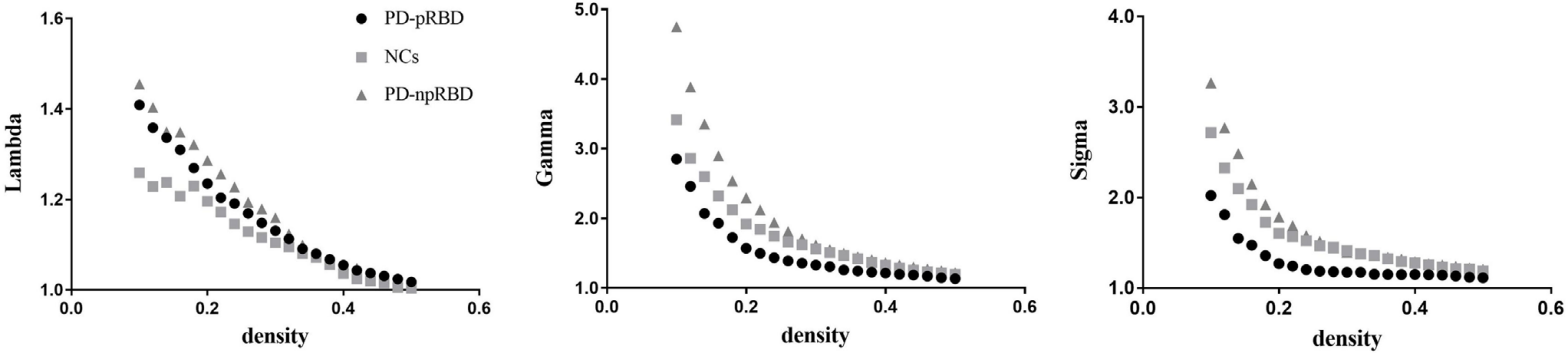
Small-worldness of normal controls (NCs), Parkinson’s disease (PD) patients with possible rapid eye movement sleep behavior disorder (RBD) (PD-pRBD), and PD patients with non-possible RBD (PD-npRBD). The graphs show the changes in Lambda, Gamma, and Sigma as a function of density thresholds. At a wide range of density, the network of each group have an average Gamma of greater than 1, an average Lambda of nearly 1, and Sigma greater than 1, which indicates prominent small-world properties.

We found that over the density range of 0.1–0.5, γ was larger than 1 and λ was near 1 (σ = γ/λ was consistently greater than 1) for all SCNs from NCs, PD-npRBD group and PD-pRBD group (Figure 2). Therefore, our study indicated that these SCNs from NCs, PD-pRBD and PD-npRBD exhibited typical features of small-worldness. No significant difference in AUC analyses of small-worldness was observed among three groups (Table 2).

**Table 2:** Areas under the curve analyses for small-world parameters and global network measures in groups.

	Controls vs. Parkinson’s disease (PD) patients with possible rapid eye movement sleep behavior disorder (RBD) (PD-pRBD) (*p* value)	Controls vs. PD patients with non-possible RBD (PD-npRBD) (*p* value)	PD-pRBD vs. PD-npRBD (*p* value)
γ	0.247	0.318	0.077
λ	0.214	0.404	0.785
σ	0.151	0.650	0.131
Global efficiency	0.563	0.791	0.713
Mean local efficiency	0.716	0.722	0.923

#### Inter-Group Comparisons of Global Network Measures

We did not find any significant difference of global network measures (global efficiency and mean local efficiency) through AUC analyses among the three groups (Table 2). This indicated that both PD-pRBD and PD-npRBD preserved global function of SCN.

#### Inter-Group Comparisons of Regional Network Measures

##### Comparisons Between NCs and PD-pRBD (PD-npRBD)

**Figure 3:**
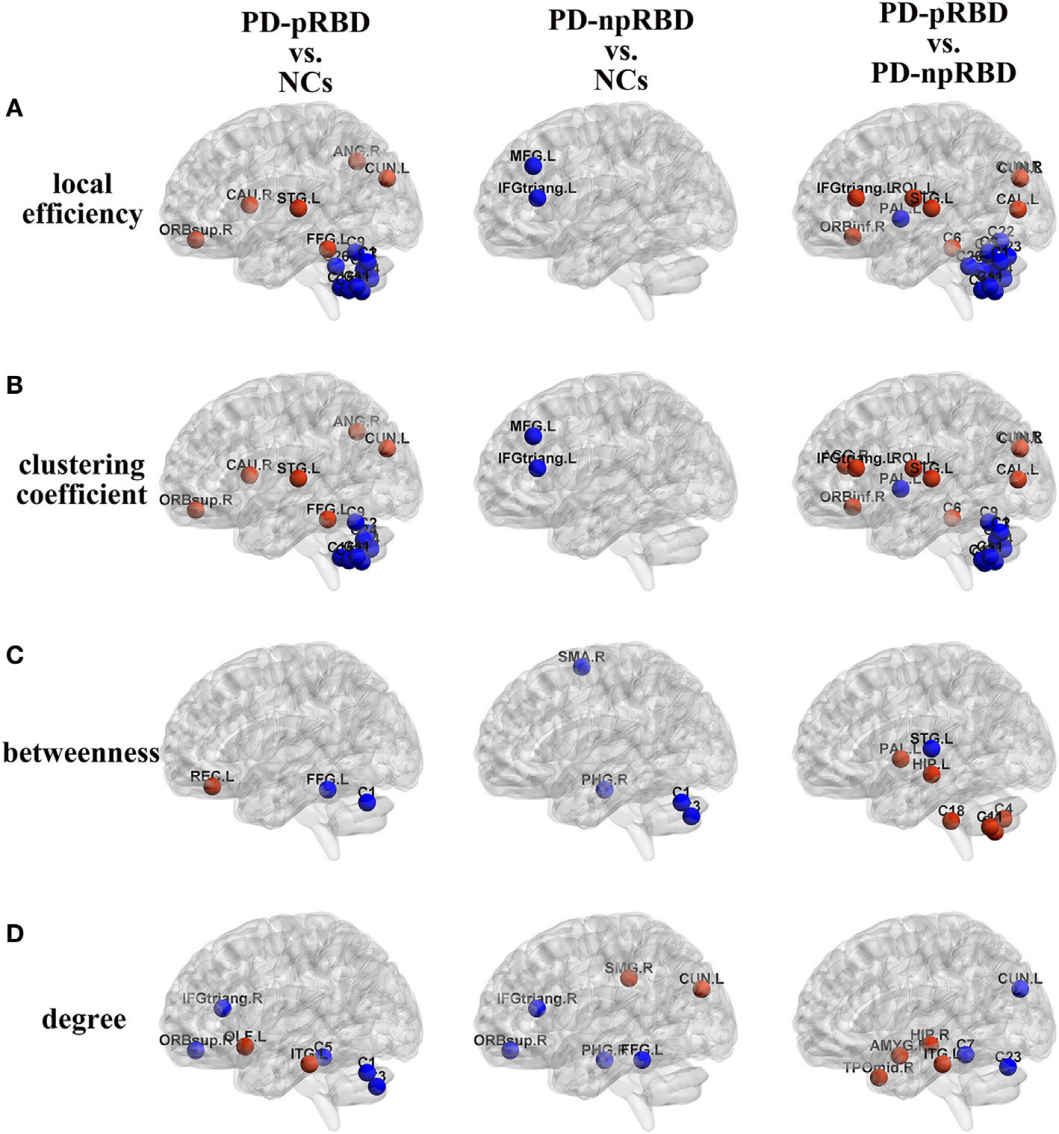
Group differences of **(A)** local efficiency, **(B)** clustering coefficient, **(C)** betweenness, and **(D)** degree between any two groups [Parkinson’s disease (PD) patients with possible rapid eye movement sleep behavior disorder (RBD) (PD-pRBD) vs. normal controls (NCs), PD patients with non-possible RBD (PD-npRBD) vs. NCs and PD-pRBD vs. PD-npRBD]. The red (blue) nodes in three panels, respectively, indicated increased (decreased) regional network measures in PD-pRBD (vs. NCs), PD-npRBD (vs. NCs), and PD-pRBD (vs. PD-npRBD). The results were visualized using the BrainNet Viewer (Beijing Normal University, http://www.nitrc.org/projects/bnv/). Abbreviations were presented in Table S3 in Supplementary Material.

In comparisons with NCs, PD-pRBD exhibited wider regions with increased nodal measures than PD-npRBD. For nodal local efficiency and clustering coefficient, PD-pRBD showed increased nodal measures in frontal-temporal regions (e.g., right pars orbitalis superior frontal gyrus, left fusiform, and left superior temporal gyrus), parietal-occipital regions (right angular and left cuneus) and right caudate. For nodal betweenness, PD-pRBD had higher betweenness in frontal region (left rectus). Besides, PD-pRBD had increased nodal degree in left olfactory and left inferior temporal gyrus. Differently, fewer regions (left cuneus and right supramarginal gyrus) with increased nodal measures were found in PD-npRBD compared with NCs (Table 3; Figure 3). In summary, when comparing with NCs, PD-pRBD showed widespread enhanced nodal properties in neocortex, whereas PD-npRBD only exhibited increased nodal degree in cuneus and supramarginal gyrus, which may indicate that presence of RBD symptoms relate to over-activation in neocortex (Table 3; Figure 3).

**Table 3:** Comparisons of nodal measures between groups.

	Local efficiency	Clustering coefficient	Betweenness	Degree
Parkinson’s disease (PD) patients with possible rapid eye movement sleep behavior disorder (RBD) (PD-pRBD) > normal controls (NCs)	Frontal_Sup_Orb_R	Frontal_Sup_Orb_R	Rectus_L	Olfactory_L
	Fusiform_L	Fusiform_L		Temporal_Inf_L
	Temporal_Sup_L	Temporal_Sup_L		
	Angular_R	Angular_R		
	Cuneus_L	Cuneus_L		
	Caudate_R	Caudate_R		

PD patients with non-possible RBD (PD-npRBD) > NCs				Cuneus_L
				SupraMarginal_R

PD-pRBD < NCs	Cerebellum_Crus1_Bi	Cerebellum_Crus1_R	Cerebellum_Crus1_L	Cerebellum_Crus1_L
	Cerebellum_Crus2_R	Cerebellum_Crus2_R	Fusiform_L	Cerebellum_Crus2_L
	Cerebellum_6_L	Cerebellum_6_L		Cerebellum_3_L
	Cerebellum_7b_Bi	Cerebellum_7b_Bi		Frontal_Inf_Tri_R
	Cerebellum_8_L	Cerebellum_8_L		Frontal_Sup_Orb_R
	Cerebellum_9_L	Cerebellum_9_L		
	Cerebellum_Vermis_8/10	Cerebellum_Vermis_8		

PD-npRBD < NCs	Frontal_Mid_L	Frontal_Mid_L	Parahippocampal_R	Parahippocampal_R
	Frontal_Inf_Tri_L	Frontal_Inf_Tri_L	Supp_Motor_Area_R	Frontal_Inf_Tri_R
			Cerebellum_Crus1_L	Frontal_Sup_Orb_R
			Cerebellum_Crus2_L	Fusiform_L

PD-pRBD > PD-npRBD	Frontal_Inf_Orb_R	Frontal_Inf_Orb_R	Hippocampus_L	Amygdala_R
	Frontal_Inf_Tri_L	Frontal_Inf_Tri_L	Pallidum_L	Hippocampus_R
	Rolandic_Oper_L	Rolandic_Oper_L	Cerebellum_Crus2_R	Temporal_Pole_Mid_R
	Calcarine_L	Calcarine_L	Cerebellum_7b_Bi	Temporal_Inf_L
	Cuneus_Bi	Cuneus_Bi	Cerebellum_10_R	
	Temporal_Sup_L	Temporal_Sup_L		
	Cerebellum_3_R	Cerebellum_3_R		
		Cingulum_Ant_R		

PD-pRBD < PD-npRBD	Cerebellum_Crus1_Bi	Cerebellum_Crus1_Bi	Temporal_Sup_L	Cerebellum_4_5_L
	Cerebellum_Crus2_R	Cerebellum_Crus2_R		Cerebellum_Vermis_7
	Cerebellum_6_Bi	Cerebellum_6_L		Cuneus_L
	Cerebellum_7b_Bi	Cerebellum_7b_Bi		
	Cerebellum_8_L	Cerebellum_8_Bi		
	Cerebellum_Vermis_6-10	Cerebellum_Vermis_8		
	Pallidum_L	Pallidum_L		

When comparing with NCs, PD-pRBD showed decreased nodal measures mainly in cerebellum, as well as in fusiform gyrus, inferior frontal gyrus and superior frontal gyrus, and PD-npRBD showed decreased nodal properties in parahippocampus, frontal areas and part of cerebellum. For detail, PD-pRBD showed decreased nodal local efficiency and clustering coefficient in bilateral cerebellum hemisphere and vermis, decreased betweenness in left cerebellum Crus1 and left fusiform, decreased nodal degree in cerebellum (left Crus1, Crus2, and cerebellum area 3), and frontal lobe (right pars triangularis inferior frontal gyrus, right pars orbitalis superior frontal gyrus). PD-npRBD showed decreased nodal local efficiency and clustering coefficient in frontal areas (e.g., middle frontal gyrus and pars triangularis inferior frontal gyrus), decreased betweenness in right parahippocampal gyrus, right supplementary motor area and left cerebellum Crus1 and Crus2, and decreased nodal degree in parahippocampal gyrus and frontal regions (pars triangularis inferior frontal gyrus, pars orbitalis superior frontal gyrus, fusiform). These results suggested that different damage patterns of brain structural network were existed in two subtypes of PD, and widespread dysfunction in cerebellum may associate with RBD symptoms (Table 3; Figure 3).

##### Comparisons Between PD-npRBD and PD-pRBD

When comparing with PD-npRBD, PD-pRBD showed increased nodal measures mainly in limbic regions, frontal-temporal regions and occipital lobe regions. In detail, PD-pRBD showed increased nodal local efficiency and clustering coefficient mainly in frontal lobe (right pars orbitalis inferior frontal gyrus, left pars triangularis inferior frontal gyrus, left Rolandic operculum) and occipital lobe (left calcarine, bilateral cuneus) as well as left superior temporal gyrus and right cerebellum area 3; increased nodal betweenness in left hippocampus gyrus, left pallidum and cerebellum (right Crus2, bilateral area 7b, and right area 10); increased nodal degree in limbic system (amygdala, hippocampal gyrus, and temporal pole); and inferior temporal gyrus. Taken together, increased nodal properties in limbic system and neocortex in PD-pRBD may suggest a potential over-activation role in these regions, which may underlie RBD (Table 3; Figure 3).

Additionally, compared with PD-npRBD, PD-pRBD exhibited decreased nodal local efficiency and clustering coefficient in bilateral cerebellum hemisphere and left pallidum, decreased betweenness in left superior temporal gyrus, and decreased nodal degree in the left cerebellum areas 4, 5, and Vermis 7 and left cuneus. In brief, decreased nodal measures mainly in cerebellum may represent the potential pathological lesion in PD-RBD (Table 3; Figure 3).

#### Hub Distribution

**Figure 4:**
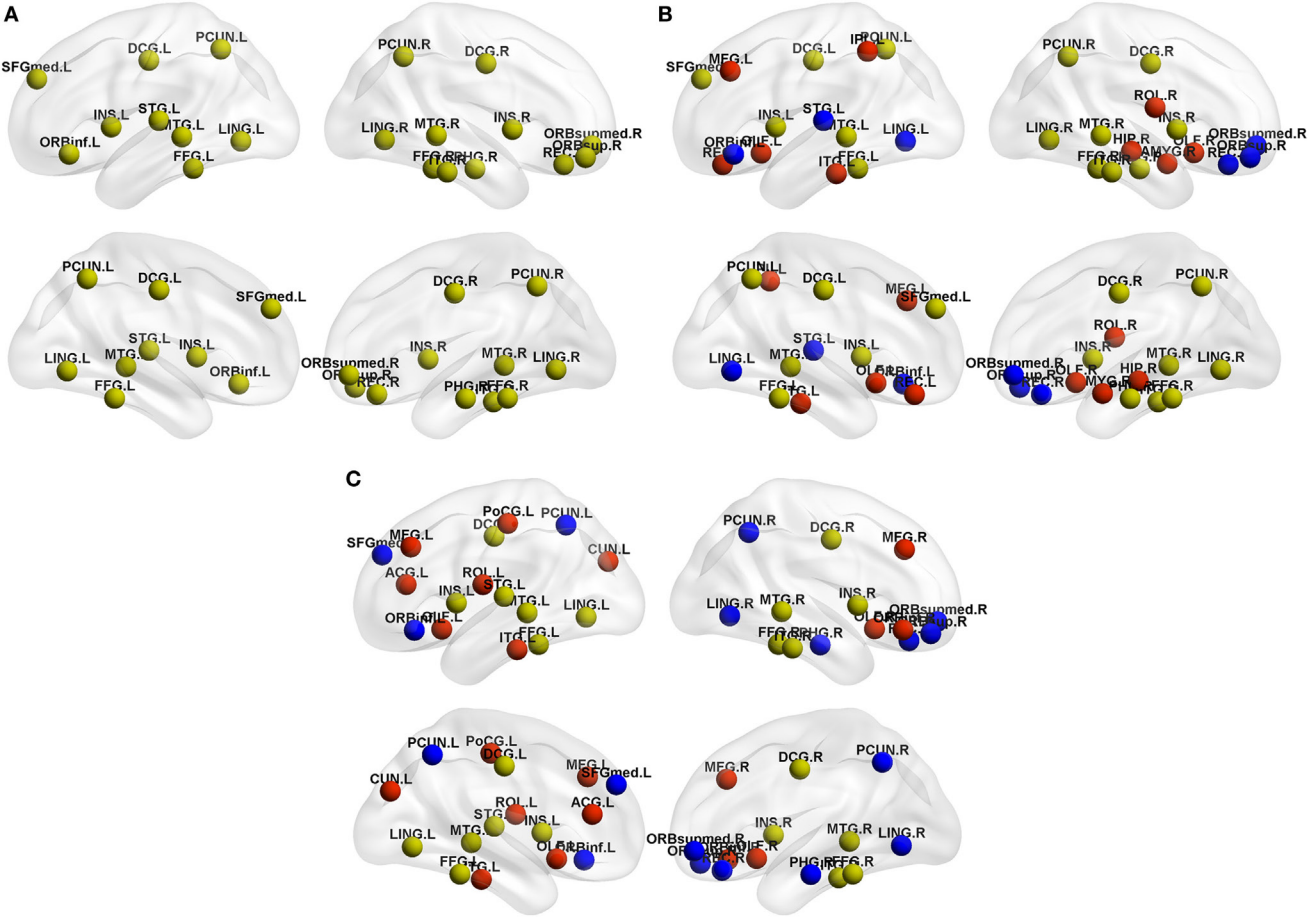
Hub distribution of the structural brain network in **(A)** normal controls (NCs), **(B)** Parkinson’s disease (PD) patients with possible rapid eye movement sleep behavior disorder (RBD) (PD-pRBD), and **(C)** PD patients with non-possible RBD (PD-npRBD). Regarding the yellow nodes in NCs as reference, red nodes in PD-pRBD and PD-npRBD groups represented the recruitment nodes, blue nodes in PD-pRBD and PD-npRBD groups represented the lost nodes. The results were visualized using the BrainNet Viewer (Beijing Normal University, http://www.nitrc.org/projects/bnv/). Abbreviations were presented in Table S3 in Supplementary Material.

As shown in Table 4, NCs had 20 regions that were identified as hubs; most of these hubs belong to frontal lobe (especially prefrontal regions), temporal lobe, limbic system, and parietal-occipital areas. PD-pRBD group had 23 regions, and PD-npRBD group had 21 regions that identified as hubs. Nine hubs, belonging temporal lobe and limbic system, co-existed in three groups. Considering the hub distribution in NCs as reference, PD-pRBD recruited eight nodes as hubs mainly located in frontal lobe and limbic system as well as left inferior temporal and left inferior parietal and lost five hubs mainly in frontal lobe as well as left superior temporal and left lingual. Compared with NCs, PD-npRBD recruited 10 nodes as hub mainly in frontal lobe as well as parietal-occipital areas and lost eight hubs mainly in frontal lobe and parietal-occipital areas as well as right parahippocampal (Table 4; Figure 4). Taken together, we could reveal two findings. First, both two PD groups exhibited reorganization of hub distribution in frontal areas. Second, specific hub alterations in PD-pRBD included recruited hubs in limbic system (amygdala, hippocampal) as well as inferior parietal gyrus and lost hubs in superior temporal gyrus as well as pars orbitalis inferior frontal gyrus, which indicated that nodes mainly in limbic system had an enhanced function accompanied by dysfunction in some other nodes. The detailed distribution of hubs is shown in Table 4 and Figure 4.

**Table 4:** Hub distribution in patients and controls.

Group	Regions	Anatomical classification	Group	Regions	Anatomical classification	Group	Regions	Anatomical classification
Normal controls (NCs)	**Co-existed hubs in three groups**		Parkinson’s disease (PD) patients with possible rapid eye movement sleep behavior disorder (RBD) (PD-pRBD)	**Co-existed hubs in three groups**		PD patients with non-possible RBD (PD-npRBD)	**Co-existed hubs in three groups**	
		
	Temporal_Inf_R	Tem		Temporal_Inf_R	Tem		Temporal_Inf_R	Tem
	Temporal_Mid_Bi	Tem		Temporal_Mid_Bi	Tem		Temporal_Mid_Bi	Tem
	Fusiform_Bi	Tem		Fusiform_Bi	Tem		Fusiform_Bi	Tem
	Cingulum_Mid_Bi	Lim		Cingulum_Mid_Bi	Lim		Cingulum_Mid_Bi	Lim
	Insular_Bi	Lim		Insular_Bi	Lim		Insular_Bi	Lim
		
	**Lost hubs in PD-pRBD or PD-npRBD**			**Co-existed hubs in NCs and PD-pRBD**			**Co-existed hubs in NCs and PD-npRBD**	
		
	Frontal_Inf_Orb_L	Fron		Frontal_Sup_Medial_L	Fron		Temporal_Sup_L	Tem
	Frontal_Med_Orb_R	Fron		Rectus_R	Fron		Lingual_L	Par-Occ
		
	Frontal_Sup_Medial_L	Fron		Parahippocampal_R	Lim		**Recruited hubs in PD-pRBD**	
		
	Frontal_Sup_Orb_R	Fron		Lingual_R	Par-Occ		Frontal_Inf_Orb_R	Fron
	Rectus_R	Fron		Precuneus_Bi	Par-Occ		Frontal_Mid_Bi	Fron
		
	Temporal_Sup_L	Tem		**Recruited hubs in PD-pRBD**			Olfactory_Bi	Fron
		
	Parahippocampal_R	Lim		Frontal_Mid_L	Fron		Rolandic_Oper_L	Fron
	Lingual_Bi	Par-Occ		Olfactory_Bi	Fron		Temporal_Inf_L	Tem
	Precuneus_Bi	Par-Occ		Rolandic_Oper_R	Fron		Cingulum_Ant_L	Lim
				Amygdala_R	Lim		Cuneus_L	Par-Occ
				Hippocampus_R	Lim		Postcentral_L	Par-Occ
				Temporal_Inf_L	Tem			
				Parietal_Inf_L	Par-Occ			

*L, left; R, right; Bi, bilateral; Fron, frontal; Tem, temporal; Lim, limbic; and Par-Occ, parietal-occipital*.

#### Robustness of Findings

After eliminating the potential effect of cognitive function on brain organization, similar alterations of network measures were found. PD-pRBD showed increased nodal local efficiency and clustering coefficient mainly in neocortex (frontal, temporal, and occipital areas), increased nodal degree in limbic system (amygdala, hippocampus, and temporal pole) as well as temporal areas, increased nodal betweenness in part of cerebellum, and decreased nodal measures in widespread cerebellum and left cuneus (results shown in Table S1 and Figure S1 in Supplementary Material). These results indicated that the network alterations mainly due to the presence of RBD symptom.

Analyses of hub distribution in PD-pRBD and PD-npRBD with covariates of age, gender, TIV, and cognitive scores showed similar hub alterations in PD-pRBD including recruited hubs in limbic system and disrupted hubs in superior temporal gyrus as well as the reorganization in frontal areas (results shown in Table S2 in Supplementary Material). These results verified that the alterations of SCN were generated by RBD symptoms.

## Discussion

In this study, we applied graph theoretical analyses to compare topology properties of GM volume-based SCN among PD groups (PD-pRBD and PD-npRBD) and NCs, which would be helpful to make out the potential neural substrates of RBD symptoms in PD. Two principal findings were observed in the present study. First, although preserved global function in two PD groups, PD-pRBD exhibited significant enhanced nodal properties in limbic system, frontal-temporal regions and occipital regions and disrupted nodal properties mainly in cerebellum. Second, PD groups exhibited both disrupt hubs and recruited hubs in frontal areas and PD-pRBD-specific hub regeneration was mainly located in limbic system.

### Preserved Global Function in PD Groups

Human brain is a formidably complex system characterized of pivotal topological properties such as small-worldness and highly centralized hubs (39, 40). Small-worldness (high clustering coefficient and short characteristic path length) is a key property of complex brain network, which supports efficient information segregation and integration with low energy and wiring costs and ensures the high rate of information transmission (35). In the present study, we found that PD-pRBD group, PD-npRBD group, and NCs group exhibited typical features of small-worldness and there was no difference between any two groups in global network measures, which indicated that both two PD groups still kept a relatively integrated global function including abilities for information processing within and across anatomically interconnected brain regions. Considering the characteristics of our PD patients (mean H&Y stage was 1.6 ± 0.5), we postulated that, at early-stage PD patients could maintain normal overall information transfer in the brain network.

### Increased Nodal Properties of Limbic-Neocortex in PD-pRBD

One of the main intriguing findings in our study was increased nodal measures in limbic regions such as amygdala, hippocampal gyrus, and temporal pole. A MRI study in iRBD showed an increased GM density in limbic regions such as hippocampi and adjacent parahippocampal gyrus, which were likely to be related to neuronal reorganization including sprouting of new connections and modification in the strength of existing connections (17, 41). Hyper-metabolism in hippocampus was also observed in iRBD patients (42). Our results of increased nodal measures in limbic system were consistent with these previous studies, which indicated that activated limbic system might relate to the pathogenesis of RBD in PD. Other studies also demonstrated sleep-related motor manifestations in RBD arise from the abnormal activation of brain structures such as limbic system (3, 43). The elaborated, complex, aberrant movements in RBD symptoms resemble as activities in awake status (44), and patients with RBD symptoms showed abnormalities in sleep-wake transitions (11), which suggested that RBD symptoms might relate to abnormal “like-arousal” status. Ascending reticular activating system (ARAS), involved in the transition from slow waves to REM sleep (45, 46), could be activated through by hippocampus and amygdala (15, 47). Therefore, we postulated that increased nodal properties in limbic system might play an over-activation role in regulation of ARAS, which promotes the level of “like-arousal,” leading to the abnormal motor behavior in REM sleep of PD patients.

Another interesting finding in our study was the increased nodal properties in frontal-temporal regions (e.g., inferior frontal gyrus, Rolandic operculum, and superior temporal gyrus) and occipital lobe regions (e.g., calcarine and cuneus and visual cortex) in PD-pRBD compared with PD-npRBD. A study using ^18^F-fluorodeoxyglucose positron emission tomography showed hyper-metabolism in frontal areas and temporal areas in iRBD (48). Furthermore, increased functional connectivity between visual cortex and pons as well as between visual cortex and frontal areas in a patient with RBD symptoms after the focal damage in pons were observed (49). Our results of increased nodal properties in frontal-temporal regions and occipital regions (visual cortex) are in line with these previous studies. Brainstem reticular formation, core pathological regions in RBD, strongly connects to frontal cortex and exhibits synchronized electrical potentials to occipital cortex (13, 50), which suggests that there are connections between brainstem reticular formation and frontal areas as well as occipital cortex. Damage in brainstem reticular formation may cause a potential effect on frontal or occipital cortex to rebalance the disrupted reticular formation, which may associate with RBD. Furthermore, ARAS could also be activated by frontal cortex and superior temporal gyrus (51) and promotes the level of “like-arousal.” The promoted ARAS may make patients more likely express the abnormal motor behavior in REM sleep. In short, increased nodal function in neocortex probably is the core pathogenesis of RBD symptoms in PD.

### Decreased Nodal Properties of Cerebellum in PD-pRBD

Besides, significantly reduced nodal properties in the various regions of cerebellum were observed in PD-pRBD. Consistently, decreased regional cerebral blood flow within cerebellar hemispheres and a volumetric decrement in the cerebellar cortex were observed in iRBD and PD-RBD (21, 52, 53), which was attributed to the impaired regional neuronal activity. Physiologically, cerebellum has a role in the regulation of the sleep-wake cycle and generation of REM atonia (54, 55). Whereas disrupted cerebellum caused by cerebellectomies leads to changes in the sleep–wake cycle and even cause sleep disorders such as RBD (54–56). Therefore, our findings were in line with previous literature that disrupts brain function in cerebellum, which provided new structural network information about the role of disrupted cerebellum in RBD.

### Plasticity of Nodal Properties in PD-pRBD

Nodal properties could be divided to two field, nodal centralities (e.g., nodal degree and nodal betweenness) and nodal cliquishness properties (e.g., nodal local efficiency and nodal clustering coefficient) (35, 36, 57). In our study, we showed a shift between nodal centralities and nodal cliquishness properties in PD-pRBD. For example, increased nodal local efficiency and nodal clustering coefficient but decreased nodal betweenness or nodal degree were observed in left superior temporal gyrus and cuneus. Similar shift were detected in left pallidum, right cerebellum Crus2 and bilateral cerebellum area 7b (increased betweenness accompanied by decreased nodal local efficiency and nodal clustering coefficient). We inferred that increased nodal centrality (nodal cliquishness properties) might balance the reduced nodal cliquishness properties (centrality), which reflect the plasticity of nodal function.

### Reorganization of Hub Distribution in PD-pRBD

Hubs are identified as nodes with a high nodal centrality (35), which indicates the importance of the nodes in the network as they interact with various brain regions (58). In our study, most hubs observed in frontal lobe, temporal lobe, limbic system (e.g., cingulate and insula cortex), as well as parietal-occipital region (e.g., precuneus) were largely consistent with the previous studies (58–63). We observed disrupt hubs both in PD-pRBD and PD-npRBD mainly located in frontal lobe. Pathologically study demonstrated that frontal lobe was a target of alpha-synuclein accumulation (10), which indicated that the common alterations in two PD groups may stem from the PD pathophysiology. Additionally, recruited hubs in frontal areas in both PD-pRBD and PD-npRBD were observed, which consist with a recent study that increased activation in frontal areas in PD (64). These findings indicated compensatory mechanism in frontal areas, which may balance the disrupted hubs in frontal areas. More importantly, PD-pRBD-specific hub regeneration was mainly located in limbic system such as amygdala, hippocampus. Recent studies revealed that iRBD patients exhibited increased metabolism in hippocampus and activated limbic system was a feature of REM sleep (13, 65), which explained the high centralities of limbic system found in the present study. Thus, reorganization of hub distribution in frontal areas may reflect the pathological damages coexisting with compensatory effects in PD. And regenerated hubs in limbic system in PD-pRBD further provided new evidence for the association between limbic system and RBD symptoms in PD.

Several limitations in this study should be addressed. First, we included PD-pRBD because polysomnography data were not included in the PPMI database. Nevertheless, RBDSQ scores used to identify RBD have a high sensitivity (96%) and specificity (85%) (32). Future studies examining the SCN in polysomnogram-verified RBD are needed. Second, some regions such as ceruleus/subceruleus complex and lateral dorsal tegmental nucleus verified to be related to RBD were not referred as nodes when constructing structural brain network due to the limited ROI extraction technology. Further studies focusing on these core pathology regions are urgent to identify the most appropriate topological structures for the brain networks of PD-RBD patients.

In conclusion, we first investigated the topological architectures of the SCNs in PD patients with and without pRBD. We found both two PD groups exhibited a remodeling of structural network. Specifically, PD-pRBD exhibited the reorganization of relatively activated brain network including enhanced nodal properties in limbic regions, frontal-temporal regions, occipital lobe regions, and recruited hubs in limbic system. Decreased nodal properties were also observed in cerebellum in PD-pRBD. These findings provided a new insight to make out the substrate of PD-RBD.

## Ethics Statement

Written informed consent was obtained from all participants, and all PPMI sites received approval from their respective ethics committee on human experimentation prior to study initiation.

## Author Contributions

All of the coauthors listed meet the criteria for authorship. TG was involved with study concept and design, preparation of data, analysis and interpretation of data, and drafting/revising the manuscript. XG, QZ, QG, and MX were involved with preparation of data, analysis, and interpretation of data. XX was involved with revising the manuscript. PH was involved with the analysis of data. MZ was involved with drafting/revising the manuscript and responsible for obtaining funding and supervision of study.

## Conflict of Interest Statement

The authors declare that the research was conducted in the absence of any commercial or financial relationships that could be construed as a potential conflict of interest.

## Abbreviations

BJLO, Benton Judgment of Line Orientation; GM, gray matter; HVLT-R, Hopkins Verbal Learning Test-Revised; LNS, letter number sequencing; NCs, normal controls; PD-pRBD, PD patients with possible RBD; PD-npRBD, PD patients with non-possible RBD; SCN, structural correlation network; SDMT, symbol digit modalities test; SF, semantic fluency.
