# Supplementary material for: Alterations of Brain Structural Network in Parkinson’s Disease With and Without Rapid Eye Movement Sleep Behavior Disorder
Source: Front Neurol. 2018 May 11;9:334. doi: 10.3389/fneur.2018.00334 (PMC5958180; doi:10.3389/fneur.2018.00334)
Supplement: Supplementary file 1 [file data_sheet_1.docx]

Supplementary Material

Alterations of Brain Structural Network in Parkinson’s Disease with and without Rapid Eye Movement Sleep Behavior Disorder

**Tao Guo, Xiaojun Guan, Qiaoling Zeng, Min Xuan, Quanquan Gu, Peiyu Huang, Xiaojun Xu, Minming Zhang^*^**

*** Correspondence:** Minming Zhang: zhangminming@zju.edu.cn

**Supplementary Methods**

**Network analyses**

***Small world parameters***

The small-worldness of a complex network has two crucial metrics: the clustering coefficient (*C_p_*) and the characteristic path length (*L_p_*). *C_p_* is taken as a measure of functional segregation and *L_p_* is taken as a measure of functional integration (1).

1. The clustering coefficient *C_i_* of a node i is defined as the number of existing links divided by the number of all possible links among the neighbors of a node:

$$C_{i}=\frac{2E_{i}}{K_{i}(K_{i}-1)}$$

where *K_i_* is the number of connections to node i, *E_i_* is the number of existing connections among the neighbors. The clustering coefficient of a network is the average of the clustering coefficient of all nodes:

$$C_{p}=\frac{1}{N}\sum_{i\in G} G_{i}$$

which is a measure of the extent of local cliquishness or local efficiency of information transfer of a network (2).

1. The shortest path length of a node in the network G (N, E) is defined as:

$$L_{i}=\frac{1}{N-1}\sum_{i\neq j\in G} d_{ij}$$

in which *d_ij_* is the shortest absolute path length between the i and j nodes. *L_p_* is the average of the shortest path length between the nodes:

$$L_{p}=\frac{1}{N}\sum_{i\in G} L_{i}$$

which quantifies the extent of average connectivity or the overall routing efficiency of the network (3).

***Network measures***

*Global measures*

Global efficiency (*E_glob_*) and mean local efficiency (*E_loc_*) were adopted to characterize the global topological organization of brain networks. Efficiency is a biologically relevant metric to describe brain networks from the perspective of parallel information flow that can deal with either the disconnected or nonsparse graphs or both (2).

1. The global efficiency *E_glob_* is defined as follows:

$$E_{glob}=\frac{1}{N(N-1)}\sum_{j\neq i\in G} \frac{1}{L_{ij}}$$

where *L_ij_* is the shortest path length between nodes i and j. *E_glob_* serves as a measure of parallel information transmission in the entire network (3).

1. The local efficiency of *G* is measured as follows:

$$E_{loc}=\frac{1}{N}\sum_{i\in G} E_{glob}(i)$$

where *E_glob_*(i) is the global efficiency of *G_i_*. *G_i_* is a subgraph that includes the nodes that connect to node i. Local efficiency measures the fault tolerance of the network, indicating the capacity for information exchange within each subgraph when the index node is eliminated (3).

*Regional nodal measures*

To examine the nodal characteristics of structural brain network, we considered four nodal metrics: nodal degree (*K_i_*), nodal betweenness (*B_i_*), nodal clustering coefficient (*C_i_*) and nodal local efficiency (*E_i_*)

1. *K_i_* represents the number of connections to a node, which is defined as follows (4):

$$K_{i}=\sum_{i\neq j\in G} e_{ij}$$

where *e_ij_* is the (i,j)th element in the binary correlation matrix.

1. *B_i_* is defined as follows(5):

$$B_{i}=\sum_{i\neq j\neq k\in G} \frac{\delta_{jk}(i)}{\delta_{jk}}$$

Where *δ_jk_* is the number of shortest paths from node j to node k and *δ_jk_*(i) is the number of shortest paths from node j to node k that pass through node i within graph *G*. *B_i_* measures the quantity of information transmitted via node i between the rest node and the entire network.

1. *C_i_* is defined as follows (6):

$$C_{i}=\frac{2E_{i}}{K_{i}(K_{i}-1)}$$

where *K_i_* is the number of connections to node i, *E_i_* is the number of existing connections among the neighbors.

1. *E_i_* is defined as follows (3):

$$E_{i}=\frac{1}{N-1}\sum_{i\neq j\in G} \frac{1}{L_{ij}}$$

where *L_ij_* is the shortest path length between nodes i and j.

**Table S1** Comparisons of regional network measures between PD-pRBD and PD-npRBD with covariates of age, sex, TIV and cognitive scores.

|  | Local efficiency | Clustering coefficient | Betweenness | Degree |
| --- | --- | --- | --- | --- |
| PD-pRBD＞PD-npRBD | Rolandic_Oper_L | Rolandic_Oper_L | Cerebellum_6_L | Amygdala_R |
|  | Calcarine_L | Calcarine_L | Cerebellum_7b_L | Hippocampus_R |
|  | Cuneus_Bi | Cuneus_Bi |  | Temporal_Pole_Mid_R |
|  | Temporal_Sup_L | Temporal_Sup_L |  | Temporal_Inf_Bi |
|  | Cerebellum_3_Bi | Cerebellum_3_Bi |  | Temporal_Mid_R |
| PD-pRBD＜PD-npRBD | Cerebellum_Crus1_Bi | \| Cerebellum_Crus1_Bi \| \| --- \| | Cuneus_L | Cuneus_L |
|  | Cerebellum_Crus2_Bi | Cerebellum_Crus2_R | Cerebellum_3_Bi | Cerebellum_Crus1_R |
|  | Cerebellum_6_Bi | Cerebellum_6_Bi |  | Cerebellum_3_Bi |
|  | Cerebellum_7b_L | Cerebellum_7b_L |  | Cerebellum_4_5_L |
|  | Cerebellum_8_L | Cerebellum_8_L |  | Cerebellum_9_R |
|  | Cerebellum_Vermis_6 | Cerebellum_Vermis_6 |  | Cerebellum_Vermis_7 |
|  | Cerebellum_Vermis_7 | Cerebellum_Vermis_7 |  | Cerebellum_Vermis_8 |
|  | Cerebellum_Vermis_8 | Cerebellum_Vermis_8 |  | Cerebellum_Vermis_9 |
|  | Cerebellum_Vermis_10 | Cerebellum_Vermis_10 |  | Cerebellum_Vermis_10 |

L = left; R = Right; Bi = Bilateral

**Table S2** Hub distribution in PD-pRBD group and PD-npRBD group

| Group | Regions | L/R/Bi | Group | Regions | L/R/Bi |
| --- | --- | --- | --- | --- | --- |
|  | Frontal lobe |  |  | Frontal lobe |  |
|  | Frontal_Mid | L |  | Frontal_Inf_Orb | R |
|  | Frontal_Sup_Medial | L |  | Frontal_Mid | Bi |
|  | Olfactory | Bi |  | Olfactory | R |
|  | Rectus | Bi |  | Rolandic_Oper | L |
|  |  |  |  | Frontal_Inf_Tri | L |
|  | Temporal lobe |  |  | Temporal lobe |  |
|  | Temporal_Inf | Bi |  | Temporal_Inf | L |
|  | Temporal_Mid | Bi |  | Temporal_Mid | Bi |
|  | Fusiform | Bi |  | Temporal_Sup | L |
| PD-pRBD |  |  | PD-npRBD | Fusiform | Bi |
|  | Limbic system |  |  | Limbic system |  |
|  | Cingulum_Mid | Bi |  | Cingulum_Ant | L |
|  | Amygdala | R |  | Cingulum_Mid | Bi |
|  | Hippocampus | R |  | Insula | Bi |
|  | Temporal_Pole_Mid | R |  |  |  |
|  | Insula | Bi |  |  |  |
|  | Parietal-occipital |  |  | Parietal-occipital |  |
|  | Lingual | R |  | Lingual | L |
|  | Precuneus | Bi |  | Cuneus | L |
|  |  |  |  | SupraMarginal | R |

L = left; R = Right; Bi = Bilateral

Table S3 Abbreviations for nodes in Figure 3 and Figure 4.

| Nodes | Abbs | Nodes | Abbs | Nodes | Abbs | Nodes | Abbs |
| --- | --- | --- | --- | --- | --- | --- | --- |
| Amygdala_R | AMYG.R | Fusiform_L | FFG.L | Rectus_L | REC.L | Cerebellum_3_L | C5 |
| Calcarine_L | CAL.L | Fusiform_R | FFG.R | Rectus_R | REC.R | Cerebellum_3_R | C6 |
| Cingulum_Ant_L | ACG.L | Hippocampus_L | HIP.L | Rolandic_Oper_L | ROL.L | Cerebellum_4_5_L | C7 |
| Cingulum_Ant_R | ACG.R | Hippocampus_R | HIP.R | Rolandic_Oper_R | ROL.R | Cerebellum_6_L | C9 |
| Cingulum_Mid_L | DCG.L | Insula_L | INS.L | Supp_Motor_Area_R | SMA.R | Cerebellum_6_R | C10 |
| Cingulum_Mid_R | DCG.R | Insula_R | INS.R | SupraMarginal_R | SMG.R | Cerebellum_7b_L | C11 |
| Cuneus_L | CUN.L | Lingual_L | LING.L | Temporal_Inf_L | ITG.L | Cerebellum_7b_R | C12 |
| Cuneus_R | CUN.R | Lingual_R | LING.R | Temporal_Inf_R | ITG.R | Cerebellum_8_L | C13 |
| Frontal_Inf_Orb_L | ORBinf.L | Olfactory_L | OLF.L | Temporal_Mid_L | MTG.L | Cerebellum_8_R | C14 |
| Frontal_Inf_Orb_R | ORBinf.R | Olfactory_R | OLF.R | Temporal_Mid_R | MTG.R | Cerebellum_9_L | C15 |
| Frontal_Inf_Tri_R | IFGtriang.R | Pallidum_L | PAL.L | Temporal_Pole_Mid_R | TPOmid.R | Cerebellum_10_R | C18 |
| Frontal_Mid_L | MFG.L | ParaHippocampal_R | PHG.R | Temporal_Sup_L | STG.L | Cerebellum_Vermis_6 | C22 |
| Frontal_Mid_Orb_R | ORBsupmed.R | Parietal_Inf_L | IPL.L | Cerebellum_Crus1_L | C1 | Cerebellum_Vermis_7 | C23 |
| Frontal_Mid_R | MFG.R | Postcentral_L | PoCG.L | Cerebellum_Crus1_R | C2 | Cerebellum_Vermis_8 | C24 |
| Frontal_Sup_Medial_L | SFGmed.L | Precuneus_L | PCUN.L | Cerebellum_Crus2_L | C3 | Cerebellum_Vermis_9 | C25 |
| Frontal_Sup_Orb_R | ORBsup.R | Precuneus_R | PCUN.R | Cerebellum_Crus2_R | C4 | Cerebellum_Vermis_10 | C26 |

**Supplementary Figure**


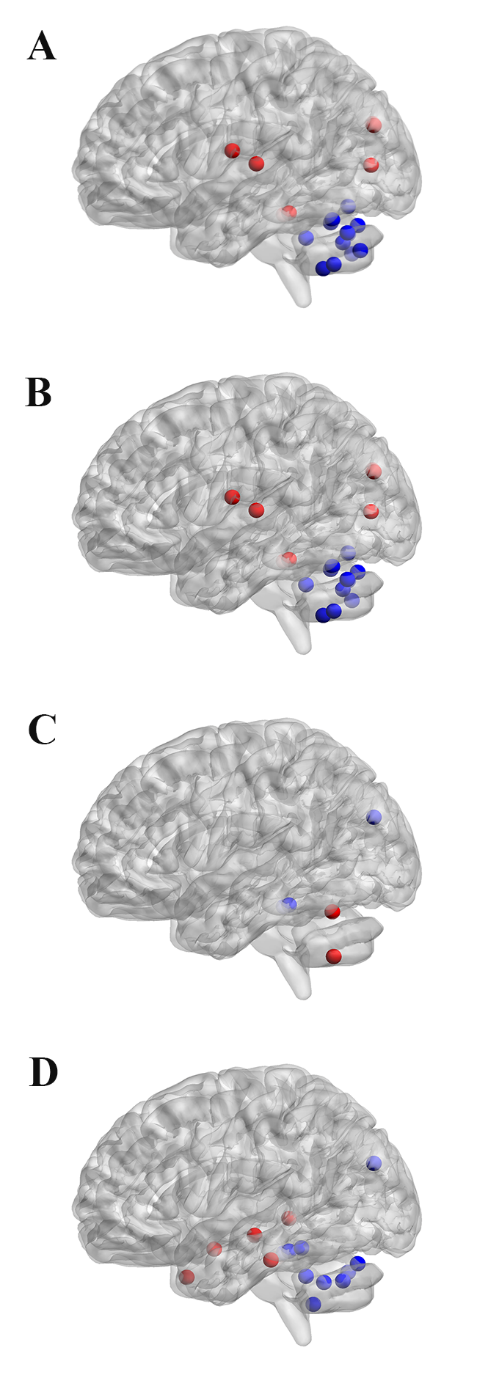


**Supplementary Figure 1**. Group differences of regional local efficiency (A), clustering coefficient (B), betweenness (C) and degree (D) between PD-pRBD group and PD-npRBD group. The red (blue) nodes indicated increased (decreased) regional network measures in PD-pRBD (vs. PD-npRBD). The results were visualized using the BrainNet Viewer (Beijing Normal University, http://www.nitrc.org/projects/bnv/).

**Reference：**

1. Watts DJ, Strogatz SH. Collective dynamics of 'small-world' networks. *Nature* (1998) 393: 440-442. doi:Doi 10.1038/30918

2. Latora V, Marchiori M. Efficient behavior of small-world networks. *Physical Review Letters* (2001) 87. doi:ARTN 19870110.1103/PhysRevLett.87.198701

3. Achard S, Bullmore E. Efficiency and cost of economical brain functional networks. *PLoS Comput Biol* (2007) 3: e17. doi:10.1371/journal.pcbi.0030017

4. Sang L, Zhang J, Wang L, Zhang J, Zhang Y, Li P, et al. Alteration of Brain Functional Networks in Early-Stage Parkinson's Disease: A Resting-State fMRI Study. *PLoS One* (2015) 10: e0141815. doi:10.1371/journal.pone.0141815

5. Freeman LC. Set of Measures of Centrality Based on Betweenness. *Sociometry* (1977) 40: 35-41. doi:Doi 10.2307/3033543

6. Xu J, Zhang J, Zhang J, Wang Y, Zhang Y, Wang J, et al. Abnormalities in Structural Covariance of Cortical Gyrification in Parkinson's Disease. *Front Neuroanat* (2017) 11: 12. doi:10.3389/fnana.2017.00012
